# Paroxetine overdose

**DOI:** 10.4103/0019-5545.55943

**Published:** 2005

**Authors:** Arun K. Gupta, Pankaj Verma, Samir K. Praharaj, Dipayan Roy, Anuradha Singh

**Affiliations:** * Senior Psychiatrist and Head, Department of Psychiatry, Dr Ram Manohar Lohia Hospital, New Delhi; ** Senior Resident, Department of Psychiatry, Dr Ram Manohar Lohia Hospital, New Delhi; *** Junior Resident, Central Institute of Psychiatry, Ranchi; **** Senior Resident, Department of Psychiatry, Dr Ram Manohar Lohia Hospital, New Delhi; ***** Senior Resident, Department of Psychiatry, Dr Ram Manohar Lohia Hospital, New Delhi

**Keywords:** Paroxetine, overdose, selective serotonin reuptake inhibitor (SSRI)

## Abstract

Paroxetine is a commonly used antidepressant with a safe side-effect profile. A case of paroxetine overdose (560 mg) is reported in an 18-year-old female who attempted suicide and recovered without any sequelae, requiring only supportive treatment. This report highlights a case of pure paroxetine overdose and the safety profile of paroxetine in overdose.

## INTRODUCTION

Paroxetine is a phenylpiperidine compound, which is the most potent inhibitor of serotonin reuptake among selective serotonin reuptake inhibitors (SSRIs). Its norepinephrine reuptake inhibition is equal to or more than that of venlafaxine. This case report highlights the safety profile of paroxetine in case of accidental overdose or a suicide attempt. To the best of our knowledge, no case of paroxetine overdose has been reported in the Indian literature.

## THE CASE

An 18-year-old unmarried female from an urban background was brought to the emergency department for attempted suicide. She had consumed 28 tablets of 20 mg paroxetine (i.e. 560 mg). She had been undergoing OPD treatment for a major depressive episode with suicidal ideation from the Department of Psychiatry, Ram Manohar Lohia Hospital, New Delhi. She had been prescribed 20 mg paroxetine daily along with 0.25 mg clonazepam, as and when required. She had no previous history of any suicidal attempt. She was regular in her medication and follow up. On her last visit, it was observed that her depressive symptoms had improved and she did not have any suicidal ideation. The suicidal attempt was an impulsive reaction precipitated by a family quarrel regarding her boyfriend. In a fit of rage, she consumed 28 tablets of 20 mg paroxetine. Three hours later, she had an episode of vomiting and subsequently, had 2 more episodes after a gap of 3 hours. The vomitus consisted of partially dissolved tablets and watery fluid.

On admission, her extremities were cold and she was perspiring. She had a pulse rate of 60/minute and her BP was 110/68 mmHg. Her body temperature was normal. Systemic examination revealed no other abnormality. She was restless and partially communicative. On mental status examination, she was conscious, was oriented to person but not to time and place; had poor attention and concentration; and was restless. Her speech was incoherent and reduced to whispering, and she did not have any delusion or hallucina-tion. An ECG revealed only sinus bradycardia, and no change in the QRS complex. The patient was kept under observation for the next 72 hours and all her medication was stopped. Only 2 L of 5% DNS was administered daily, and no other intervention was required. Her pulse, BP and cardiovascular status were monitored after every hour and ECG recorded twice a day. Her vitals improved within 48 hours, with the pulse rate becoming 74/minute and BP 128/78 mmHg. She started taking food orally. On day 3, the mental status examination showed that her cognition had improved, she was well oriented to time, place and person, and had a fair level of attention and concentration. Her memory was intact, psychomotor activity was normal, speech was coherent and goal-directed. The patient was depressed, had a feeling of guilt and had grade V insight.

## DISCUSSION

Paroxetine is relatively safe in overdose as compared to tricyclic antidepressants (TCAs) and is rarely fatal when taken alone.^1^ Patients have survived paroxetine overdoses of up to 3600 mg.^2^ Moderate overdoses of SSRI (up to 30 times the normal daily dose) have been found to be associated with minor or no symptoms. The intake of a large amount usually results in drowsiness, tremor, nausea, and vomiting; very high doses of SSRIs (>75 times the normal daily dose) may result in seizures, ECG changes and decreased consciousness.^1^ Symptoms of paroxetine overdose are nausea, vomiting, facial flushing, sedation, dizziness and sweating; and at very high doses, myoclonus, hyperreflexia and seizures are seen.^3^ Most patients described in the literature who have had features of severe serotonin syndrome due to paroxetine overdose were receiving other drugs as well.^2^,^4^ Cases of pure paroxetine overdose are few in the literature. This case highlights the features of pure paroxetine overdose, i.e. nausea, vomiting, cold and clammy extremities, bradycardia and a fall in the BP, disorientation, restlessness and incoherence of speech. Most patients recover with conservative management and require little intervention as in this case. Deaths involving paroxetine toxicity have generally been a result of ingestion of multiple drugs, and not from paroxetine alone.^5^,^6^ Hyponatraemia has been reported with paroxetine in the elderly.^7^ Paroxetine overdose (360 mg of paroxetine) was reported in an octagenaerian, who responded to fluid restriction, sodium chloride infusion and levothyroxine.^8^ In a review of SSRI overdose, no agent was found superior to the other with respect to safety in overdose.^1^ This case further supports the safety of paroxetine in overdose and supports the current preference for SSRIs over TCAs in the treatment of depression.

## References

[1] Barbey JT, Roose SP (1998). SSRI safety in overdose. J Clin Psychiatry.

[2] Velez LI, Shepherd G, Roth BA (2004). Serotonin syndrome with elevated paroxetine concentrations. Ann Pharmacother.

[3] Bourin M, Chue P, Guillon Y (2001). Paroxetine: A review. CNS Drug Rev.

[4] FitzSimmons CR, Metha S (1999). Serotonin syndrome caused by overdose with paroxetine and moclobemide. J Accid Emerg Med.

[5] Goeringer KE, Raymon L, Christian GD (2000). Postmortem forensic toxicology of selective serotonin reuptake inhibitors: A review of pharmacology and report of 168 cases. J Forensic Sci.

[6] Vermeulen T (1998). Distribution of paroxetine in three postmortem cases. J Anal Toxicol.

[7] Azaz-Livshits T, Hershko A, Ben-Chetrit E (2002). Paroxetine associated hepatotoxicity: A report of 3 cases and a review of the literature. Pharmacopsychiatry.

[8] Johnsen CR, Hoejlyng N (1998). Hyponatremia following acute overdose with paroxetine. Int J Clin Pharmacol Ther.

